# Supplementary material for: Prognostic value of syntax score, intravascular ultrasound and near-infrared spectroscopy to identify low-risk patients with coronary artery disease 5-year results from the ATHEROREMO and IBIS-3 cohorts
Source: PLoS One. 2022 Nov 10;17(11):e0275592. doi: 10.1371/journal.pone.0275592 (PMC9648722; doi:10.1371/journal.pone.0275592)
Supplement: S1 Appendix — (DOCX) [file pone.0275592.s001.docx]

APPENDIX

| **Table 1** 1 year FU | **Unadjusted model** | **p-value** | **Adjusted for  Age & Gender** | **p-value** | **Adjusted for  Age, Gender & indication CAG** | **p-value** |
| --- | --- | --- | --- | --- | --- | --- |
| Syntax ≤8 | HR 0.67 (0.40-1.12) | 0.1 | HR 0.69 (0.41-1.15) | 0.2 | HR 0.68 (0.40-1.13) | 0.1 |
| PB ≤70 | HR 0.47 (0.28-0.78) | 0.003* | HR 0.51 (0.31-0.85) | 0.009* | HR 0.51 (0.31-0.85) | 0.010* |
| LCBI_4mm_ ≤227 | HR 0.32 (0.16-0.64) | 0.001* | HR 0.33 (0.16-0.65) | 0.001* | HR 0.32 (0.16-0.64) | 0.001* |
|  | | | | | | |
| Syntax ≤8   + PB ≤70 | HR 0.73 (0.44-1.23) HR 0.50 (0.66-1.92) | 0.2 0.007* | HR 0.74 (0.44-1.25) HR 0.54 (0.32-0.90) | 0.263 0.018* | HR 0.73 (0.43-1.23) HR 0.54 (0.32-0.90) | 0.239 0.018* |
| Syntax ≤8  + LCBI_4mm_≤227 | HR 0.62 (0.37-1.04) HR 0.32 (0.16-0.65) | 0.067 0.001* | HR 0.63 (0.38-1.06) HR 0.33 (0.17-0.66) | 0.083 0.002* | HR 0.63 (0.37-1.06) HR 0.33 (0.16-0.66) | 0.079 0.002* |
| PB ≤70  + LCBI_4mm_ ≤227 | HR 0.55 (0.33-0.92) HR 0.34 (0.20-0.52) | 0.023* 0.003* | HR 0.59 (0.35-0.99) HR 0.35 (0.17-0.70) | 0.047* 0.003* | HR 0.60 (0.36-1.00) HR 0.35 (0.17-0.69) | 0.052 0.003* |
|  |  |  |  |  |  |  |
| Syntax ≤8  + PB ≤70  + LCBI_4mm_ ≤227 | HR 0.67 (0.40-1.13) HR 0.60 (0.35-1.01) HR 0.35 (0.17-0.71) | 0.1 0.056 0.003* | HR 0.68 (0.40-1.15) HR 0.64 (0.38-1.08) HR 0.35 (0.18-0.71) | 0.2 0.095 0.003* | HR 0.68 (0.40-1.15) HR 0.64 (0.38-1.09) HR 0.35 (1.17-0.70) | 0.1 0.1 0.003* |
